# Supplementary material for: Relating sparse and predictive coding to divisive normalization
Source: PLoS Comput Biol. 2025 May 27;21(5):e1013059. doi: 10.1371/journal.pcbi.1013059 (PMC12112309; doi:10.1371/journal.pcbi.1013059)
Supplement: S1 Appendix — This document has some further analysis of the work (PDF) [file pcbi.1013059.s001.pdf]

S1 Appendix

1 Choice of $H(\cdot)$

In the original papers of sparse coding [1] and predictive coding [2], the choices of $Q(s)$ considered were

$$Q(s) = \ln\left[1 + \left(\frac{s}{\sigma}\right)^2\right] \ , \quad -e^{-(\frac{s}{\sigma})^2} \ , \ |s| \ \text{ or } \ s^2 \ , \tag{A1}$$

where σ is a positive scaling factor. With the assumption of nonnegative responses, we consider here the three different functions for $Q(s)$:

$$Q(s) = \ln\left[1 + (\frac{s}{\sigma})^2\right] \quad -e^{-(\frac{s}{\sigma})^2} \ , \quad \text{or} \quad s^\alpha, \tag{A2}$$

where α is a positive power index. The corresponding homeostasis functions are $\lambda Q'(s)$, namely:

$$H(s) = \lambda \frac{s}{1 + (s/\sigma)^2} \ , \quad \lambda s e^{-(\frac{s}{\sigma})^2} \quad \text{or} \quad \lambda s^{\alpha - 1} \quad (\text{with } s \geq 0) \ , \tag{A3}$$

where the constant parameters are absorbed into the single constant parameter, λ.

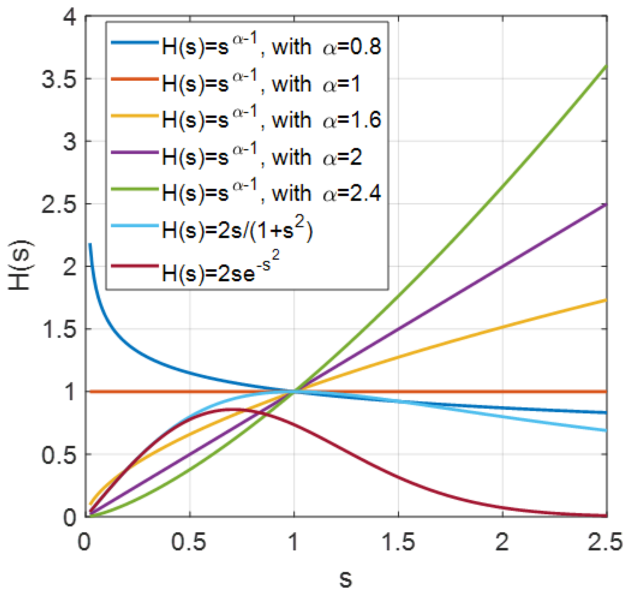

Fig-A 1: **Examples of homeostasis functions.** These functions are examples of Eq. A3. λ is set to 1 for power index functions. For the remaining functions, λ is set to 2 and σ is set to 1 so that all functions can be plotted in the same range.

Some examples of homeostasis functions, $H(\cdot)$, are shown in Fig. 1. For power functions, when $H(s) = 1$ ($\alpha = 1$), the cost the homeostasis puts on model neurons is the same for different firing rates; when α is smaller than 1, the cost of generating small firing rates is extremely high (infinite cost for zero firing rate) and the cost decreases dramatically for larger firing rates, i.e., when $\alpha > 1$, the cost of generating firing rates starts from 0 and increases without bound as the firing rate increases. For $H(s) = 2s/(1 + s^2)$ and $H(s) = 2se^{-s^2}$, the cost starts from zero, reaches the maximum and then reduces to zero as the firing rate increases. Overall, the choice of $H(\cdot)$ is crucial because it partly determines the dynamics of the model, as described by Eq. 10.

2 When $H(s) \neq 0$

If there is a homeostatic fixed point of the activity, namely a value of the firing rate $\mathbf{s}^{\mathrm{EQ}}$ such that $H(\mathbf{s}^{\mathrm{EQ}})$ is non-zero, then generally the equilibrium value $\mathbf{s}^{\mathrm{EQ}}$ does not have an explicit analytical formula, except for some special choices of $H(\cdot)$ such as $H(s) = c$ where c is a constant.

If $H(s) = c$, Eq. 13 becomes

$$-\mathbf{A}^T\mathbf{A}\mathbf{s}^{\mathrm{EQ}} + \mathbf{A}^T\mathbf{x} = \mathbf{c}, \tag{A4}$$

where $\mathbf{c}$ is a vector whose elements are all equal to c. Similar to the analysis above, the equilibrium, $\mathbf{s}^{\mathrm{EQ}}$ can then be written as

$$\mathbf{s}^{\mathrm{EQ}} = (\mathbf{A}^T\mathbf{A})^{-1}(\mathbf{A}^T\mathbf{x} - \mathbf{c}), \tag{A5}$$

when $\mathbf{A}^T\mathbf{A}$ is invertible.

For other more complex choices of $H(\cdot)$, an explicit formula for the analytical equilibrium has not been found, though its value maybe determined from the intersection between $Y(\mathbf{s}) = -\mathbf{A}^T\mathbf{A}\mathbf{s} + \mathbf{A}^T\mathbf{x}$ and $H(\mathbf{s})$, as show in Fig. 3.

3 When $H(s) = 0$

If there is no homeostasis (i.e., $H(s) = 0$), Eq. 13 becomes

$$\mathbf{A}^T\mathbf{A}\mathbf{s}^{\mathrm{EQ}} = \mathbf{A}^T\mathbf{x}. \tag{A6}$$

Since $\mathbf{A}^T\mathbf{A}$ is positive semi-definite, $\mathbf{A}^T\mathbf{A}$ will be invertible (i.e., positive definite) if we assume $\mathbf{A}\mathbf{z} \neq \mathbf{0}$ for any vector $\mathbf{z} \neq \mathbf{0}$. This condition is generally met for sensory system in the brain, since there will be neurons responding to a non-zero input stimulus. The equilibrium, $\mathbf{s}^{\mathrm{EQ}}$ can then be written as

$$\mathbf{s}^{\mathrm{EQ}} = (\mathbf{A}^T\mathbf{A})^{-1}\mathbf{A}^T\mathbf{x}. \tag{A7}$$

The equilibrium of SPC model with no homeostasis is still linear, but the equivalent feedforward connection becomes $(\mathbf{A}^T\mathbf{A})^{-1}\mathbf{A}^T$. In addition, the amplitude of the model responses will increase linearly with the amplitude of the input. If $\mathbf{A}^T\mathbf{A} = \mathbb{I}$, the equilibrium of the SPC model will be identical to the response of a linear feedforward model with input $\mathbf{x}$ and feedforward connection weight matrix $\mathbf{A}^T$. Given that the dynamic equations Eq. 12 describe a first order differential equation, the model responses, $\mathbf{s}$, will approach the equilibrium, $\mathbf{s}^{\mathrm{EQ}}$, as

$$\mathbf{s}(t) = [\mathbf{v}_1 \ \mathbf{v}_2 \ \cdots \mathbf{v}_M] \begin{bmatrix} \mathbf{v}_1^T\mathbf{s}(0)e^{\lambda_1 t} \\ \mathbf{v}_2^T\mathbf{s}(0)e^{\lambda_2 t} \\ \vdots \\ \mathbf{v}_M^T\mathbf{s}(0)e^{\lambda_M t} \end{bmatrix} + \mathbf{s}^{\mathrm{EQ}} \ , \tag{A8}$$

where $\mathbf{v}_i$ $(i = 1, 2, \cdots, M)$ are the eigenvectors and λ_i $(i = 1, 2, \cdots, M)$ are the corresponding eigenvalues of $-\mathbf{A}^T\mathbf{A}$, and $\mathbf{s}(0)$ is the initial response at time 0. Because $-\mathbf{A}^T\mathbf{A}$ is negative definite with the assumptions above, λ_i $(i = 1, 2, \cdots, M)$ will be all negative, meaning that the first term on the right hand side of Eq. A8 will reduce to zero exponentially.

Figs. 2 & 3 show the simulation results to validate the analysis above when there is no homeostasis in the model. In the simulation there are 256 units in the first layer ($L = 256$) and 256 units in the second layer ($M = 256$). Therefore $\mathbf{A}$ is a 256×256 matrix and each column is a basis vector used to reconstruct the input image. The input is a 16×16 image of sinusoidal grating (Fig. 2B). There are 3000 iterations of Eq. 10 to compute the model responses with $\eta_s = 0.05$ and there is no learning process of $\mathbf{A}$ in this simulation. $\mathbf{A}$ is chosen differently for Figs. 2 and 3.

The connection $\mathbf{A}$ in Fig. 2 is chosen as

$$\mathbf{A} = \mathbb{I} + \mathbb{N} \ \text{ with } \ \|\mathbf{A}\|_2 = 1 \ , \tag{A9}$$

where $\mathbb{I}$ is an 256×256 identity matrix and $\mathbb{N}$ is an 256×256 matrix whose elements are sampled from a Gaussian distribution with mean 0 and standard deviation 0.05. $\mathbf{A}$ is then normalized to have l_2 norm 1. $\mathbf{A}$ is chosen this way to ensure that $\mathbf{A}^T\mathbf{A}$ is a full-rank matrix and the eigenvalues of $\mathbf{A}^T\mathbf{A}$ are all different but separated from 0. Fig. 2A displays the 256 basis vectors and these vectors are used to reconstruct the input image (Fig. 2B). Fig. 2C shows that the model response becomes stable within

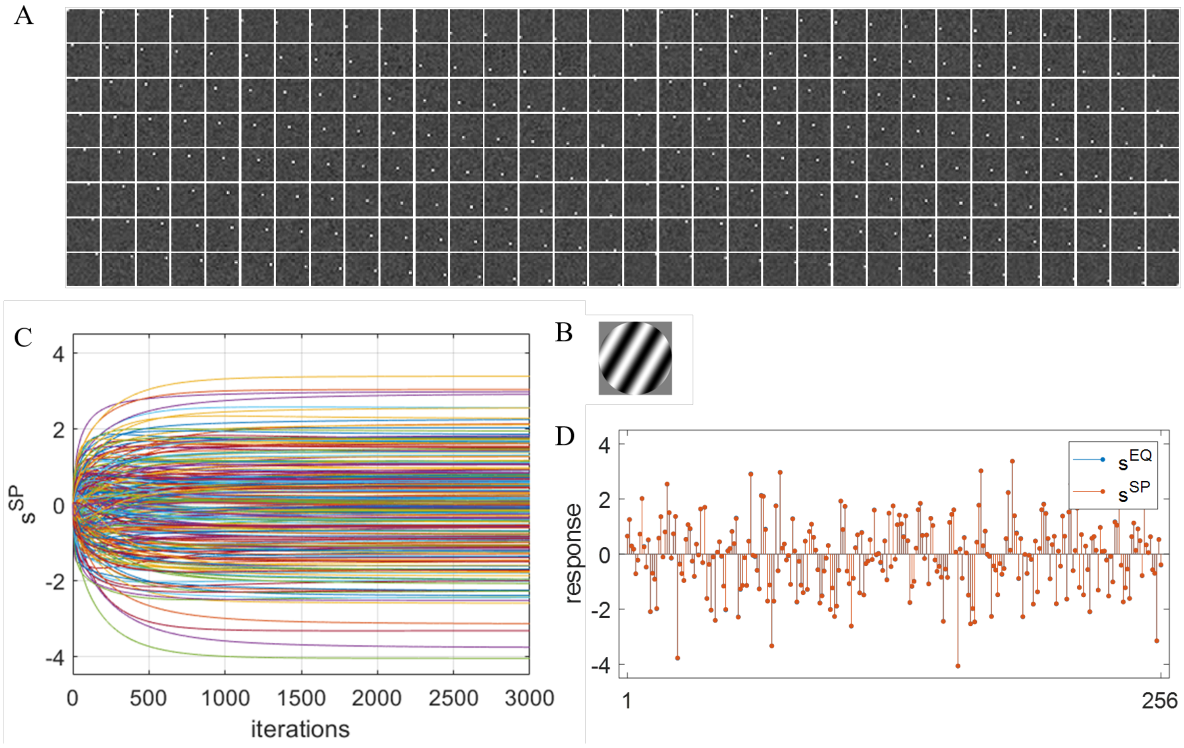

Fig-A 2: **Model response converges to the analytical equilibrium.** There is no homeostasis in this simulation. (A) Basis vectors of $\mathbf{A}$. Each square block is a 16×16 image. $\mathbf{A}$ is defined in Eq A9 and close to an identity matrix, so each basis vector has one dominant element. (B) The 16×16 input image. (C) Response trajectory. (D) The response of analytical equilibrium and response of SPC model after 3000 iterations of computing model responses with $\eta_s = 0.05$.

3000 iterations. The analytical equilibrium $\mathbf{s}^{\mathrm{EQ}}$ (Eq. A7) is plotted as red spikes and the model response $\mathbf{s}^{\mathrm{SP}}$ after 3000 iterations is plotted in blue spikes in Fig. 2D. Fig. 2D shows that the model response is identical to the analytical equilibrium. The summed squared error between $\mathbf{s}^{\mathrm{EQ}}$ and $\mathbf{s}^{\mathrm{SP}}$ is only 0.0036% and the summed squared error between input ($\mathbf{x}$) and reconstruction ($\mathbf{As}$) is 0. In other words, given the current input and fixed connection, the model can essentially represent the input perfectly and the model response converges to the analytical value.

Although the model has an analytical equilibrium described by Eq. A7, it does not guarantee that model response will converge to the analytical equilibrium within any reasonable time. On the contrary, the iterated model response might be far away from the equilibrium while the model response can still reconstruct the input very well, which is illustrated in Fig. 3.

In Fig. 3, the connection weights $\mathbf{A}$ are chosen to be some Gabor-like filters that are learnt by a standard sparse coding model [1, 3, 4] and $\|\mathbf{A}\|_2 = 1$. Fig. 3A shows the 256 basis filters of $\mathbf{A}$, which look similar to simple cells found in the visual cortex [5]. Though $\mathbf{A}^T\mathbf{A}$ is invertible with all positive eigenvalues, some eigenvalues are very small (the smallest is 4×10^{-10}). Therefore, the analytical response described by Eq. A7 may become very large. This is illustrated in Fig. 3B, which shows that the analytical equilibrium is quite large and the amplitude in this example can grow as large as 500. Fig. 3C shows the trajectory of model response over these 3000 iterations of implementing model dynamics with $\eta_s = 0.05$ (the same as Fig. 2). However, it appears that model responses do not converge after 3000 iterations. The model responses after 3000 iterations are plotted in Fig. 3D. Compared with the analytical equilibrium shown in Fig. 3B, the amplitude of model response is much smaller than the analytical equilibrium. Apart from the different amplitudes of responses, the analytical equilibrium and model response also differ in the population responses, which are illustrated in Fig. 3E, that shows that the responses are very different even after normalizing the responses to the same scale. The summed squared error between the equilibrium and model responses is 99.99%. The difference between analytical and simulated results may be caused by the discrete implementation of the model and the very small eigenvalues of $\mathbf{A}^T\mathbf{A}$, which takes much longer for the model response to converge, as described by Eq. A8. Despite the significant difference between the analytical equilibrium and model simulation response, the model can still reconstruct the

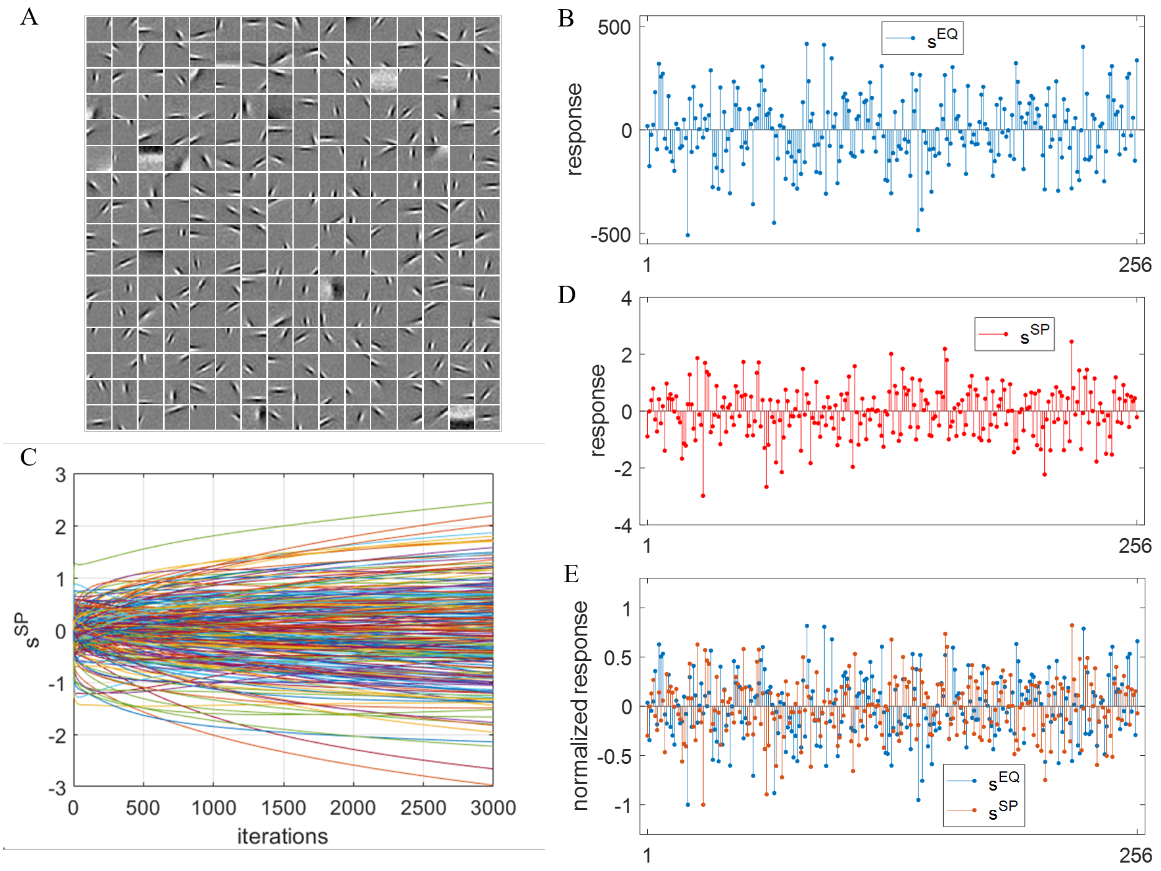

Fig-A 3: **Model response does not converge to the analytical equilibrium.** There is no homeostasis in this simulation and the input to the model is the same as Fig. 2B. (A) Basis vectors of $\mathbf{A}$. Each square block is a 16×16 image. $\mathbf{A}$ is learned using a standard sparse coding model and each basis filter is similar to simple cells in V1. (B) $\mathbf{s}^{\text{EQ}}$, the analytical equilibrium determined by Eq. A7. (C) Response trajectory. (D) Model response, $\mathbf{s}^{\text{SP}}$, after 3000 iterations. (E) The normalized response of analytical equilibrium and model response after 3000 iterations with $\eta_s = 0.05$.

input well, with a small summed squared error of 0.8% and the average energy of the model response (mean of the squared response) is 0.77. However, if there are only 300 iterations, the reconstruction error of the model is 1.5% and the average energy is 0.21. Therefore, there seems to be a trade-off between the model accuracy and computation cost (including both time and energy). In practice, although $\mathbf{A}^T\mathbf{A}$ is mostly invertible, some of the eigenvalues can easily be very small, as illustrated in Fig. 3. Also, if the standard deviation of $\mathbb{N}$ in Eq. A9 becomes 0.1, the smallest eigenvalues of $\mathbf{A}^T\mathbf{A}$ would be 9.9×10^{-7}. Therefore, though the discrete online implementation of the model is not as accurate as the analytical result, it nevertheless provides a reasonable response within a reasonable time.

4 Nonlinearity

The SPC model implemented in our simulations have non-negative firing rates. The nonlinearity introduced into the SPC model is two-fold: through the nonnegativity of the firing rates and through the homeostasis function.

As analyzed in the previous section, the equilibrium of the SPC model is linearly dependent on the input when there is no constraint of nonnegative firing rate and homeostasis (Eq. A7), given that $\mathbf{A}^T\mathbf{A}$ is invertible. However, $\mathbf{s} \geq \mathbf{0}$ has a dramatic effect on the network, because this constraint introduces great nonlinearity. Somewhat surprisingly, the nonnegativity constraint accelerates the convergence of the model responses and it becomes stable after 1500 iterations (Fig. 4A) compared with the case without the nonnegativity constraint (Fig. 3C). However, because of the nonnegativity of $\mathbf{s}$, the model cannot reconstructs the input with negative responses though the basis vectors $\mathbf{A}$ in Fig. 3 are learned in a

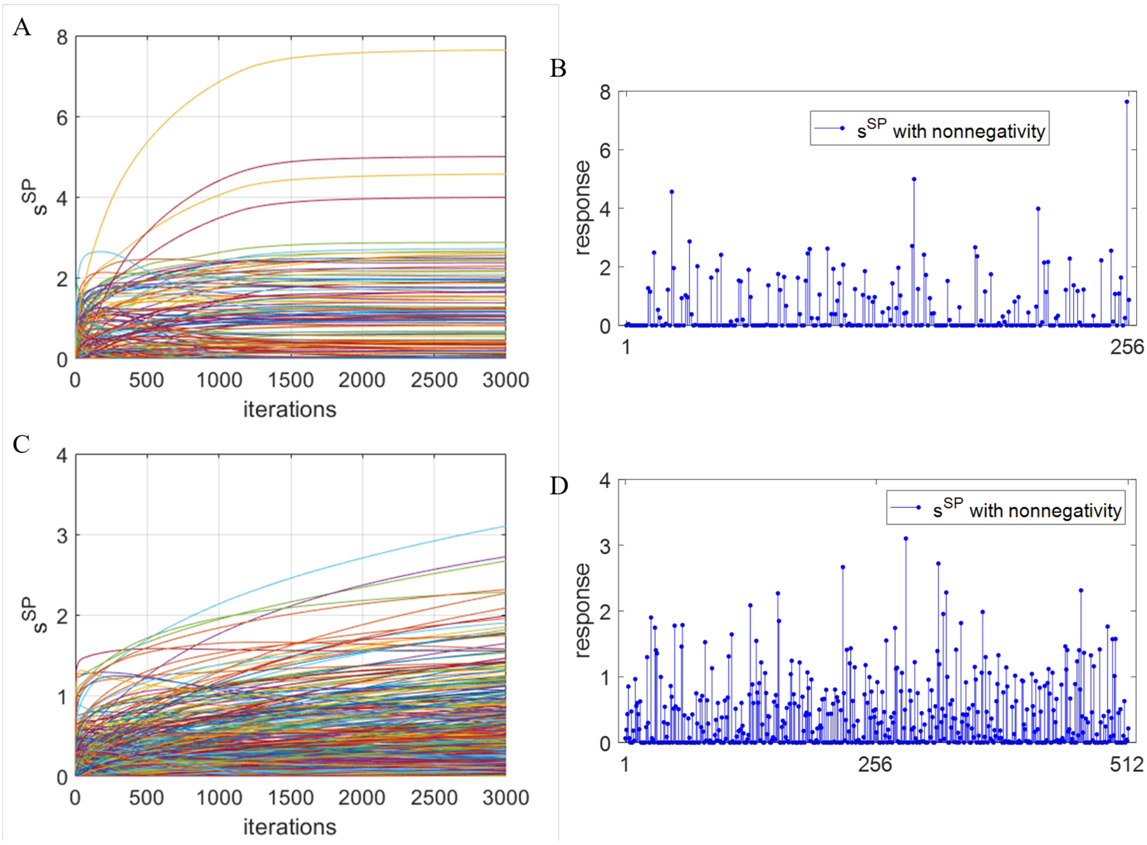

Fig-A 4: **Model responses with nonnegative constraint.** The input to the model is the same as Fig. 2B. (A, C) Response trajectory. (B, D) $\mathbf{s}^{\mathrm{SP}}$, after 3000 iterations. For (A) and (B), the same basis vectors $\mathbf{A}$ as in Fig. 3A are used. For (C) and (D), the basis vectors are extend to be $[\mathbf{A}\ \ -\mathbf{A}]$ and the number of model units also doubles, so there are 512 model units in (C) and (D).

standard sparse coding that allows model responses to be negative, which leads to a larger reconstruction error of 11.1% after incorporating $\mathbf{s} \geq \mathbf{0}$. Fig. 4B illustrates the model responses after 3000 iterations when $\mathbf{s} \geq \mathbf{0}$ is incorporated and it shows a clear difference from the analytical response ($\mathbf{s}^{\mathrm{EQ}}$ in Fig. 3B) and the model responses without the nonnegativity constraint (Fig. 3D). After the nonnegativity of model response is incorporated, one way to preserve the expressiveness of the basis vectors is to extend the basis vectors to be $[\mathbf{A}\ \ -\mathbf{A}]$; i.e., similar to a previous study [6]. Therefore, there are 512 model units, instead of 256 model units in this case. Figs. 4C & D show that the convergence of model units is lost again and more model units are active, which leads to a better reconstruction of the input with 0.8% error.

Apart from the nonnegativity of the model response, the homeostasis, $H(s)$, also introduces nonlinearity into the model, since the system becomes nonlinear if $H(s)$ is not a first-order polynomial of s. Even when there is no nonnegative constraint on the model response, the choice of $H(s)$ can determine the model responses and ultimately determines whether the model can learn structures from the input. For example, only suitable choices of sparsity functions can lead to Gabor-like simple cells after learning [1, 2, 3].

5 Competition

SPC model incorporates competition into the neural network via the homeostasis function and the model structure.

The homeostasis function, $H(\cdot)$ in Eq. 10, introduces competition because it directly regulates the response of model units and it can put different penalties on different units based on their response level.

Apart from the homeostasis function, the structure of SPC model itself (Fig. 2) has a competitive nature because it explicitly implements a form of competition, as demonstrated in a previous study [7]. Suppose there is no homeostasis (i.e., $H(s) = 0$) in the dynamic equation of SPC, Eq. 10 essentially becomes the implementation of Harpur's "negative feedback network" [8] that causes competition between

model units such that they compete to represent the input. That competition can also be seen from the analytical equilibrium described in Eq. A7, where $(\mathbf{A}^T\mathbf{A})^{-1}$ will always scale the linear response $(\mathbf{A}^T\mathbf{x})$ based on the correlation between different basis vectors. However, the competition incorporated by this structure can be very strong such that learning complex cells from simple cells can be very challenging [9].

Because of this competitive nature of SPC model, sparse coding has been used to explain some nonclassical receptive field properties of V1 such as end-stopping, contrast invariance of orientation tuning [6]. However, this study failed to explain contrast saturation, which is another important property of V1 neurons.

References

[1] B. A. Olshausen and D. J. Field, "Sparse coding with an overcomplete basis set: A strategy employed by V1?," *Vision Res.*, vol. 37, no. 23, pp. 3311–3325, 1997.

[2] R. P. Rao and D. H. Ballard, "Predictive coding in the visual cortex: a functional interpretation of some extra-classical receptive-field effects," *Nat. Neurosci.*, vol. 2, no. 1, pp. 79–87, 1999.

[3] B. A. Olshausen and D. J. Field, "Emergence of simple-cell receptive field properties by learning a sparse code for natural images," *Nature*, vol. 381, no. 6583, pp. 607–609, 1996.

[4] C. J. Rozell, D. H. Johnson, R. G. Baraniuk, and B. A. Olshausen, "Sparse coding via thresholding and local competition in neural circuits," *Neural Comput.*, vol. 20, no. 10, pp. 2526–2563, 2008.

[5] D. H. Hubel and T. N. Wiesel, "Receptive fields of single neurones in the cat's striate cortex," *J. Physiol.*, vol. 148, no. 3, pp. 574–591, 1959.

[6] M. Zhu and C. J. Rozell, "Visual nonclassical receptive field effects emerge from sparse coding in a dynamical system," *PLoS Comput. Biol.*, vol. 9, no. 8, p. e1003191, 2013.

[7] M. W. Spratling, "Reconciling predictive coding and biased competition models of cortical function," *Front. Comput. Neurosci.*, vol. 2, p. 4, 2008.

[8] G. F. Harpur and R. W. Prager, "Development of low entropy coding in a recurrent network," *Network: Comput. Neural Syst.*, vol. 7, no. 2, pp. 277–284, 1996.

[9] Y. Lian, A. Almasi, D. B. Grayden, T. Kameneva, A. N. Burkitt, and H. Meffin, "Learning receptive field properties of complex cells in V1," *PLoS Comput. Biol.*, vol. 17, no. 3, p. e1007957, 2021.
